# Reconstructive Paradigms: A Problem-Solving Approach in Complex Tissue Defects

**DOI:** 10.3390/jcm13061728

**Published:** 2024-03-17

**Authors:** Andreea Grosu-Bularda, Florin-Vlad Hodea, Andrei Cretu, Flavia-Francesca Lita, Eliza-Maria Bordeanu-Diaconescu, Cristian-Vladimir Vancea, Ioan Lascar, Serban Arghir Popescu

**Affiliations:** 1 “Carol Davila” University of Medicine and Pharmacy Bucharest, 050474 București, Romania; andreea.grosu-bularda@umfcd.ro (A.G.-B.); ioan.lascar@umfcd.ro (I.L.); serban.popescu@umfcd.ro (S.A.P.); 2 Clinic of Plastic Surgery and Reconstructive Microsurgery, Clinical Emergency Hospital Bucharest, 011602 București, Romania; 3 Clinic of Plastic Surgery and Reconstructive Microsurgery, Central Military Universitary Emergency Hospital “Carol Davila”, 010825 București, Romania

**Keywords:** reconstructive surgery, paradigm, decision-making, therapeutic strategies

## Abstract

The field of plastic surgery is continuously evolving, with faster-emerging technologies and therapeutic approaches, leading to the necessity of establishing novel protocols and solving models. Surgical decision-making in reconstructive surgery is significantly impacted by various factors, including the etiopathology of the defect, the need to restore form and function, the patient’s characteristics, compliance and expectations, and the surgeon’s expertise. A broad surgical armamentarium is currently available, comprising well-established surgical procedures, as well as emerging techniques and technologies. Reconstructive surgery paradigms guide therapeutic strategies in order to reduce morbidity, mortality and risks while maximizing safety, patient satisfaction and properly restoring form and function. The paradigms provide researchers with formulation and solving models for each unique problem, assembling complex entities composed of theoretical, practical, methodological and instrumental elements.

## 1. Introduction

Plastic surgery is a specialized branch of surgery addressing the reconstruction, repair and improvement of the human body. Although some plastic surgery techniques have been practiced since antiquity, this specialty gained popularity in World War I, when surgeons were called upon to reconstruct the post-traumatic facial and limb defects of wounded infantrymen. Between the wars, further subspecialties of plastic surgery developed, one being the area of craniofacial surgery. World War II then accelerated the technological development process of plastic surgery with newly developed technical abilities, highlighting the need for emerging paradigms in the field [1,2,3,4].

In recent decades, technology has determined major changes in the paradigms of the medical field. In plastic surgery and reconstructive microsurgery, a major afflux of new information and innovation occurred, resulting in a broad range of reconstructive options [5,6].

Elaboration of reconstructive paradigms has eased the decisional process for each plastic surgeon in choosing the optimal reconstructive method for each particular case. Reconstructive surgery often presents multiple possible surgical techniques for the same case, providing different simultaneous approaches, and raising challenges in making the therapeutic decision [7,8].

## 2. Overview of Reconstructive Paradigms

Various reconstructive paradigms have emerged, each primarily aimed at effectively addressing the surgical needs of each specific case. The concept of paradigm in the scientific field was proposed by Thomas Kuhn in the book “The Structure of Scientific Revolutions”, in the year 1962. “Paradigms are scientific realizations, that are universally recognized, for a period, that offer a problem-solution model to a community of scientific practitioners” [9,10].

**Figure 1:**
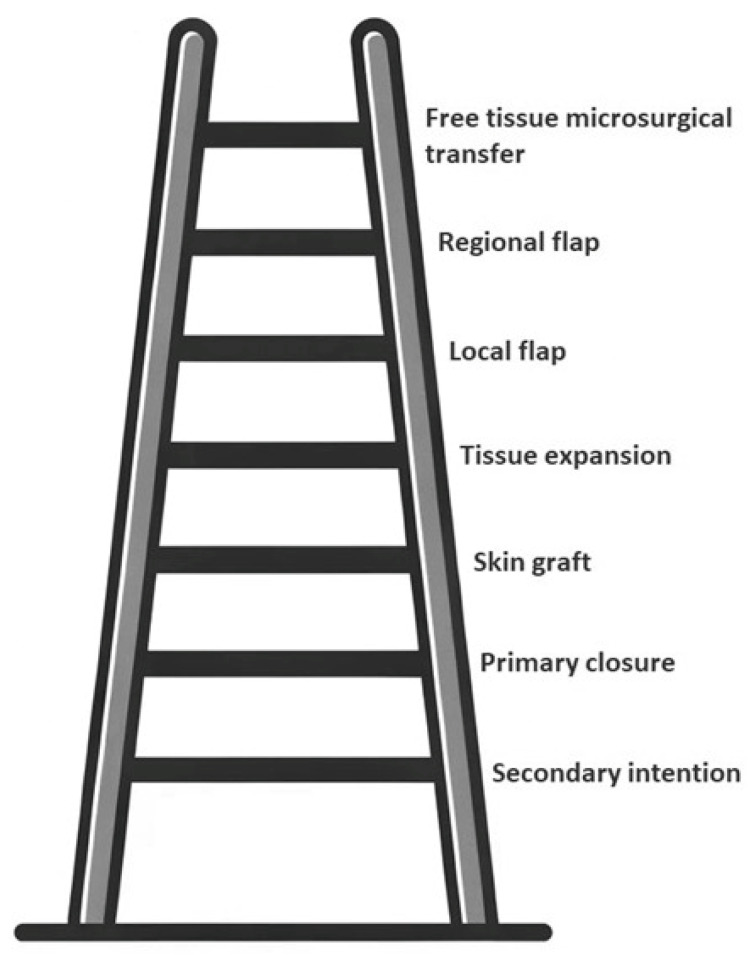
The reconstructive ladder (adapted after [16,17]).

First described by Sir Harold Gillies, the concept of the “reconstructive ladder” was further popularized by Mathes and Nahai, allowing the surgeon to choose the procedure from simple to complex [11,12]. The reconstructive ladder established reconstructive priorities based on required technical complexity when approaching a defect or deformation, aiming to obtain the safest result for the patient [13,14,15]. Figure 1 displays the concept of the reconstructive ladder [16,17].

Based on this concept, it is advised to prioritize the use of the simplest reconstructive technique that can cover the tissue defect. The disadvantage of this approach is that form and function restoration is not necessarily optimal by choosing the simplest technique that ensures defect coverage. Complex reconstructions with a functional aim require a complex intervention, and in these cases, decisions must be taken early during patient therapy, hence the reason for the next proposed reconstructive paradigm [18,19,20].

This different approach was proposed by Gottlieb and Krieger in 1994, who described the “reconstructive elevator”, stating that the surgeon must choose the most adequate reconstructive “floor” respectively, the most adequate technique, regardless of its complexity, which ensures the best functional result.

Therefore, in some cases, the correct approach would be choosing a complex reconstructive procedure in the first place, including microvascular flap transfers (as opposed to the concept of the successive simple to complex approach, as promoted by the reconstructive ladder) [18,21,22].

Another reconstructive paradigm is the “reconstructive triangle” proposed by Mathes and Nahai in 1997, integrating the three most important reconstructive options: tissue expansion, utilization of flaps, and lastly microsurgical techniques. This means choosing the safest surgical intervention for the patient that ensures adequate form and function restoration while minimizing donor site morbidity, emphasizing a more tailored approach to each patient’s needs and the specifics of his condition [18,23,24,25].

Another approach is that of “the reconstructive matrix” described by Erba et al.; a tridimensional cubic model comprising three axes, respectively, technological refinement, surgical complexity, and patient’s surgical risk. Therefore, a “reconstructive medium” is defined with an infinite number of points representing all potential approaches to a tissue defect, also taking into consideration socio-economic factors. In this virtual space, a dynamic interaction between the three axes is generated, and a continuously expanding matrix of potential reconstructive interventions is established. This approach aims to generate new and efficient solutions tailored to patient’s problems [26].

**Figure 2:**
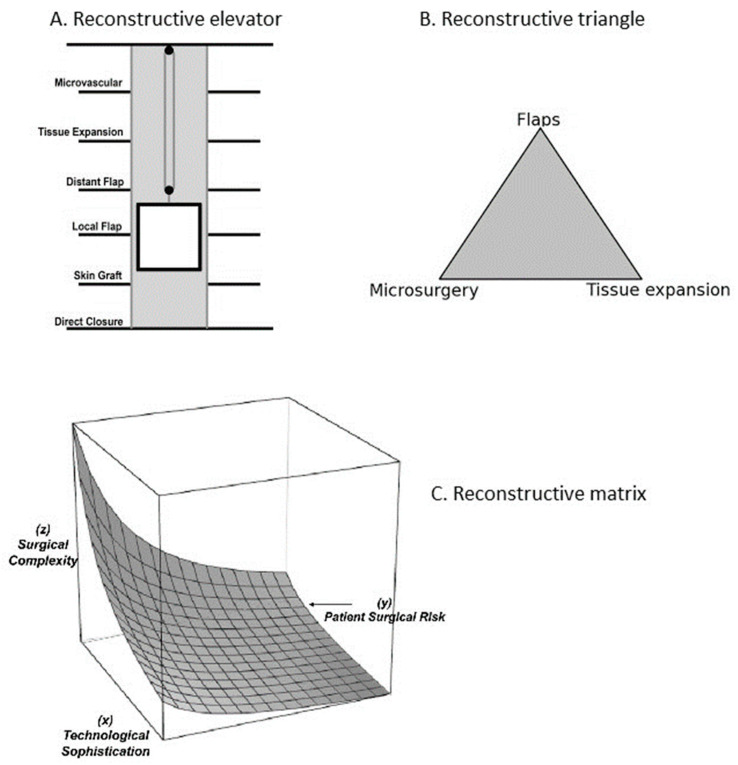
(**A**) The reconstructive elevator. (**B**) Reconstructive triangle. (**C**) Reconstructive matrix (adapted after [19,21,23,26]).

The concepts of reconstructive elevator, triangle and matrix are illustrated in Figure 2.

Wong and Niranjan proposed the concept of “reconstructive stages”. While going through the steps of this concept, emphasis is put on the development of surgical abilities, underlying that the difficulty of a reconstructive problem is directly dependent on the surgeon’s experience and skill. The surgeon gradually gains experience, sequentially progressing from simple procedures to highly complex ones, such as free flap microsurgery [27].

**Figure 3:**
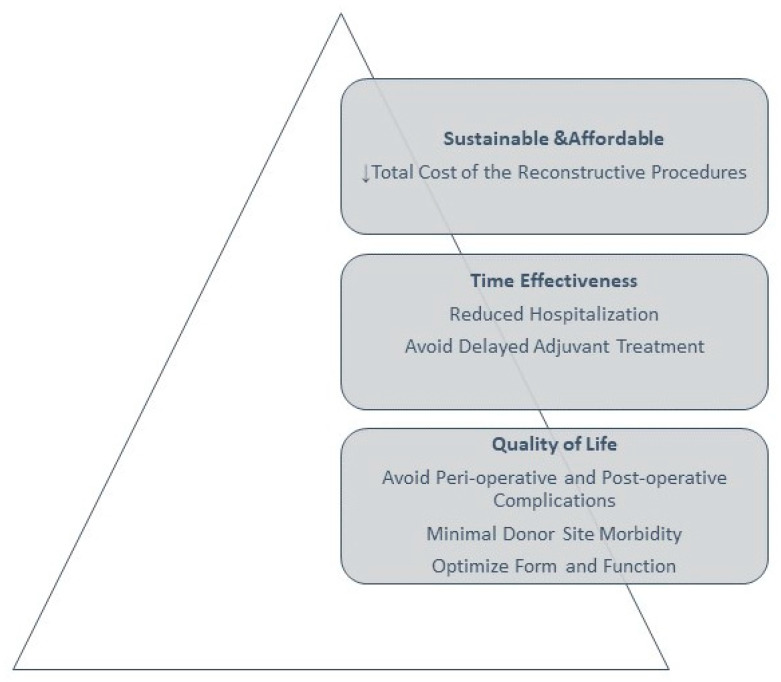
Hierarchical pyramid (adapted after [7]).

In response to the existing limitations in choosing the correct reconstructive procedures, Al Deek and Wei have proposed an alternative concept, that of the reconstructive pyramid. A hierarchical pyramid approach focusing on reconstructive objectives, as seen in Figure 3, can serve as a model for creating a customized algorithm for a specific region or structure, such as the face, breast, hand or lower extremity. The foundation of this pyramid should be the improvement of quality of life and building upwards from there, while the approach would prioritize delivering timely and cost-effective treatment. The effectiveness of these methods should be confirmed through patient-reported outcomes, focusing on goals that go beyond mere wound coverage, aiming instead to efficiently and cost-effectively restore structure and function, thereby enhancing the overall quality of life for patients [7].

New therapeutic entities have been emerging lately, with important reconstructive potential, such as skin substituents, negative pressure therapy combined with other interventions, tissue bioengineering for soft tissues, new flap models (perforator flaps, “free-stye” flaps, prefabricated and prelaminated flaps), and vascularized composite allotransplantations. It is mandatory to include these new strategies in the therapeutical options armamentarium in order to provide optimal solutions to patients, increasing results structurally, functionally and esthetically. The aim is to regain or enhance previous quality of life parameters and patient social and professional reintegration as soon as possible [20,25,28].

The “translational reconstructive ladder” concept proposed by De Francesco is an integration of regenerative medicine techniques and conventional reconstructive methods. This innovative approach is particularly effective for treating large wounds with significant soft tissue damage, ensuring improved and lasting healing. It is also applicable in various other contexts, such as pain management, inflammatory and chronic diseases, vascular disorders, post-cancer reconstruction and more. Additionally, translational medicine incorporates a range of advanced techniques, including the use of cell and tissue engineering scaffolds, a decellularized extracellular matrix, wearable medical devices, micro- and nanomedicine, 3D bioprinting, biomimetic engineering, organ-on-a-chip technologies, and bioelectronics [13].

Regenerative medicine is revolutionizing the field of plastic surgery, introducing groundbreaking approaches that enhance healing and improve outcomes. This emerging discipline focuses on repairing, replacing, or regenerating human cells, tissues, or organs to restore or establish normal function. In plastic surgery, the principles of regenerative medicine are being applied through innovative techniques such as tissue engineering, the application of adult stem cell therapy (ASCs), and the utilization of growth factors for optimal healing, including platelet-rich plasma (PRP) [29,30,31,32,33,34].

An important field is represented by the introduction into the clinical practice of mesenchymal stem cells due to their promising regenerative potential. Mesenchymal stem cells may be isolated from bone marrow, adipose tissue and umbilical cord blood [35]. Human adipose-derived stem cells (ADSCs) are a type of multipotent autologous mesenchymal stem cells. Known for their versatility, these adult stem cells seem to play a significant role in a broad spectrum of therapeutic applications across reconstructive surgery and various other medical fields, where they could offer substantial potential in healing and regeneration [35,36]. Gir et al. reviewed a series of clinical applications of cell therapies using (ADSCs) in various pathologies, such as digestive diseases: Crohn’s disease fistulas; fecal incontinence; autoimmune disorders; diabetes mellitus type 1 and type 2, cardiovascular diseases: nonrevascularizable myocardium; ST-segment elevation myocardial infarction; chronic limb ischemia; peripheral vascular disease, and neurologic diseases: spinal cord injury; secondary progressive multiple sclerosis; skeletal regeneration in degenerative arthritis, with potential in the development of bone grafts. Also, the same paper identifies clinical trials using autologous adipose-derived stem cell transplantation in the field of plastic surgery for different problems such as lipodystrophy, Romberg disease, depressed scars, wound healing and as a tool in breast reconstruction and soft tissue augmentation [37].

In recent decades, fat grafting has become a popular technique in plastic surgery for treating a variety of conditions, due to its benefits such as biocompatibility, affordability, minimal complications, and reduced morbidity to donor sites [38,39]. The problems associated with lipofilling reconstruction are the unpredictability of the results, variability in fat survival rate and fat necrosis [40]. To address the challenges associated with lipoinjection, different strategies were introduced. One of the methods, the cell-assisted lipotransfer (CAL) involves the use of autologous adipose-derived stem (stromal) cells (ASCs) in conjunction with lipofilling. By isolating a stromal vascular fraction (SVF) that includes ASCs from a part of the harvested fat and then mixing it with the remaining fat, this technique effectively enhances the originally ASC-deficient aspirated fat with a higher concentration of ASCs [41,42]. In a systematic review published by Li, it was shown that cell-assisted lipotransfer improves fat survival rates in breast augmentation in comparison with conventional lipofilling [43]. In 2013, Tonnard et al. introduced the “nanofat” concept, which is a product derived from the lipoaspirate tissue through a process of emulsification and filtration, having as its purpose a stimulating effect in tissue regeneration through stem cells regeneration [44]. Since then, the use of nanofat increased significantly for various applications like improving lipofilling results in tissue augmentation and specific fields as breast reconstruction, the treatment of pathologic scars after trauma or burns, wound healing, the regeneration of tendons and ligaments, bone and cartilage repair and also facial aesthetics procedures [45]. The combination of nanofat with platelet-rich plasma boosts the growth and the differentiation into an adipogenic lineage with superior results in cosmetic procedures and also for wound healing indications [45,46].

Mesenchymal stem cells (MSCs) derived from bone marrow also offer promising therapeutic potential for tissue regeneration and repair [47]. These adult stem cells, characterized by their ability to differentiate into various cell types including bone, cartilage, and fat cells, are promising in developing advanced treatments for a range of conditions [48]. In reconstructive surgery, bone marrow-derived MSCs are utilized for their regenerative potential for wound healing, the repair of bone and cartilage defects, and the restoration of skin and soft tissue defects [48,49,50]. Their immunomodulatory properties also make them valuable in reducing inflammation and promoting tissue regeneration, influencing the microenvironment through cytokines and growth factors release [47,51,52]. Furthermore, the use of MSCs in reconstructive procedures has been associated with improved healing times, reduced scarring, and enhanced functional outcomes, marking a significant advance in the ability to restore form and function following injury or surgical intervention [53,54].

Tissue engineering exemplifies a key aspect of this paradigm shift in plastic surgery. It represents a multidisciplinary approach to creating functional biological tissues, involving the use of scaffolds and various cellular types to construct three-dimensional tissue-like structures. These methods not only aid in more effective wound healing and scar reduction, but also open new avenues for reconstructive surgeries, such as breast reconstruction post-mastectomy, complex skin substitutes, bio-engineered compounds for composite tissue repair and nerve regeneration [55,56,57,58].

The association of stem cells and structural scaffolds permits the creation of three-dimensional complex media, active from a biological standpoint with numerous physiochemical properties [59]. The scaffolds play the role of the supportive framework that guides the growth and organization of the cells into tissues, ultimately mimicking the extracellular matrix. These constructs hold immense potential for regenerative medicine, with research ongoing to explore innovative materials for optimizing scaffold design and, ultimately, for achieving the best compatibility with the human body [30,60,61,62,63,64].

These three-dimensional scaffold structures are made from biocompatible materials and are designed to mimic the natural extracellular matrix, providing a framework for cell growth and differentiation. They are used to reconstruct damaged tissues, such as skin, bone, and cartilage. Advances in fabrication techniques, including 3D printing, allow for the creation of scaffolds that are tailored to specific patient defects, enhancing integration with native tissue [65,66]. Scaffolds can be divided into natural polymers or inorganics and synthetic polymers or inorganics. Some examples of scaffolds include carbon nanotube and methyl-methacrylate for bone substitution, or poly (l-lactide-co-ε-caprolactone) and polyglycolic acid for vascular conduits. Adding nanofillers such as silver or gold nanoparticles may determine a higher mechanical strength of scaffolds, better stability, and improved cell adhesion, proliferation and differentiation. In addition, growth factors may be delivered in scaffolds in order to promote cell activity towards a desired pathway [67]. The combination of scaffolds with growth factors and stem cells is pushing the boundaries of reconstructive surgery, offering more effective and personalized treatment options. Recent research focuses on the development of complex structures combining specific scaffolds with anti-tumor drugs, in order to provide a controllable drug delivery method, with great potential for suppressing tumoral growth. Mei et al. describe porous polylactic acid/methotrexate (PLA/MTX) scaffolds obtained using three-dimensional printing technology as drug delivery devices to reduce tumor growth [68]. Transdermal and topical drug delivery systems are also proposed, with particular relevance in skin cancer treatment. Utilizing hydrogel-based formulations for delivering antiproliferative agents in melanoma skin cancer offers numerous benefits compared to traditional drug delivery methods and conventional treatments [69]. The use of hydrogels is highly favored for their superior biocompatibility, biodegradability, and efficient drug encapsulation and release capabilities. They may be incorporated into various cancer treatments, including radiotherapy, chemotherapy, immunotherapy, hyperthermia, photodynamic therapy, and photothermal therapy [70].

In reconstructive surgery, tissue engineering addresses the restoration of various anatomical components, using the aforementioned recent technologies, with particular applications. In the field of nerve repair, recent research has extended beyond enhancing nerve regeneration strategies to the development of various nerve conduits. The objective has been to identify materials that elicit a minimal immune response, possess strong mechanical qualities, and promote the regenerative process. These conduits aim to match the effectiveness of autologous nerve grafts, providing similar results and functional outcomes in a cost-effective manner. Progress has been noted with the use of collagen, polysaccharides, and synthetic polymers in designing nerve conduits [71,72,73,74].

There are a variety of different skin substitutes that can be used in plastic surgery, but they all have the advantage of promoting wound healing through an extracellular matrix. Skin substitutes can be allografts or xenografts, depending on their human or animal origin, respectively, but they might also be made from biosynthetic materials. They are useful especially when large defects need to be covered and there are not enough donor sites for autografts. In these situations, skin substitutes represent both a physical barrier against germs and a proper environment for wound healing. They mimic the structure and function of normal skin, promoting tissue healing and minimizing scarring, playing a crucial role in treating burns and chronic wounds [75,76,77,78,79].

Negative Pressure Wound Therapy (NPWT) is a technique that uses a vacuum dressing, being largely used both as a pillar in the management of complex wounds and as a promoter of tissue repair postoperatively. It aids in wound healing by promoting blood flow and preventing surgical-site complications such as infections and wound dehiscence. Thus, in chronic wounds such as diabetic ulcers or pressure ulcers, granulation tissue formation might be achieved faster through NPWT [80,81,82,83,84]. Negative pressure wound therapy proves highly beneficial when used in conjunction with skin grafts, as it enhances graft adherence to the recipient’s wound bed and promotes vascular integration of the grafts for improved healing [82,85].

The emphasis on technological integration, particularly in craniofacial surgery, and the focus on addressing current challenges and future perspectives, highlight the dynamic and evolving nature of reconstructive and plastic surgery as we move further into the 21st century [25,86].

In the past decades, modern imaging techniques have advanced the field of medical diagnostics and treatment planning, providing great insights into anatomical structures. Some of the more widely used are angiographic computed tomography (angio-CT) or angiographic magnetic resonance imaging (angio-MRI), which are appropriate imagistic techniques for describing vascular structures such as blood vessels, vascular abnormalities or variants and the structural modifications existent in complex tissular defects [87,88,89,90].

On the other hand, another tool in diagnostics and pre-surgical planning is emerging 3D printing technology. It allows the creation of physical models from data collected from imaging studies, which enables surgeons to have a tangible representation of intraoperative findings. Three dimensional printing technologies also provide a combination of volumetric analysis and production of biological materials that can be used for scaffolding in the tissue engineering process [91,92,93].

A category of defects that are difficult to reconstruct are the complex, three-dimensional ones, which also have significant functional implications. Such situations are frequently encountered in the craniofacial region. One of the most challenging aims of head and neck reconstruction was and still is multiple component reconstruction, combining functional soft tissue areas with structural support. The introduction of new concepts, like “Jaw in a Day” reconstruction, revolutionizes maxillofacial surgery by enabling the complete restoration of the jaw within a single surgical session, a task that traditionally might require multiple surgeries over an extended time frame. This approach combines the removal of the affected bone with the immediate reconstruction using pre-planned, custom-made implants and grafts, facilitated using advanced 3D imaging and printing technologies, respectively the CAD/CAM (computer-aided design/computer-aided manufacturing) technology. Free scapula, fibula, or iliac crest grafts are used for bony reconstruction. It often includes the placement of dental implants and provisional prostheses, allowing patients to leave with both functional and aesthetic restoration in just one day. This method significantly reduces recovery time and improves the quality of life by minimizing the physical and emotional impact of jaw reconstruction, showcasing a remarkable advancement in patient care through the integration of multidisciplinary expertise [94,95,96,97,98].

In addition, technologies of AR (augmented reality) have emerged in planning the surgical case of complex head and neck cases. This may lead to another paradigm shift by using augmented reality planning tailored to each patient, which superposes 3D angiographic tomography with patient topography, enhancing planning and decision making, and having proved to also improve outcomes [99]. In situations where the case is too complex and conventional methods are not feasible, the tridimensional reconstruction of the head and neck area employs techniques such as prefabrication and prelamination, combined with tissue engineering and stem cells [100].

Microsurgery is the subfield of plastic surgery that has enabled significant progress in surgical techniques and also the incorporation of new and revolutionary technologies within the specialty, as those previously mentioned. In the early 1990s, Levin introduced as a new paradigm a specialized reconstructive ladder, exclusively focusing on microsurgical techniques [101].

Over the past six decades, the field of microsurgery has undergone a significant evolution, beginning with the revascularization and replantation techniques and then extending to the utilization of free microsurgical transfer for the remediation of tissue defects and the implementation of composite tissue transfer methods. A notable development is the concept of chimeric flaps and the application of functional muscle transfers to address motor deficits following brachial plexus injuries or Volkman’s ischemic contracture, progressing further to the realm of hand transplantation [12,102].

Furthermore, enhanced comprehension of cutaneous vascular anatomy, particularly the angiosome concept, has facilitated the development of perforator flaps and freestyle flaps. These innovative techniques have significantly reduced the necessity for sacrificing major vascular pedicles, such as the radial artery or terminal arteries [12,103,104,105,106,107].

Additionally, the emergence of supermicrosurgical techniques has been pivotal, especially in the microsurgical management of lymphedema. In this context, the introduction and application of microsurgical robotic surgery have shown promising outcomes, offering new perspectives and approaches in this complex and challenging field [108,109,110,111,112].

**Figure 4:**
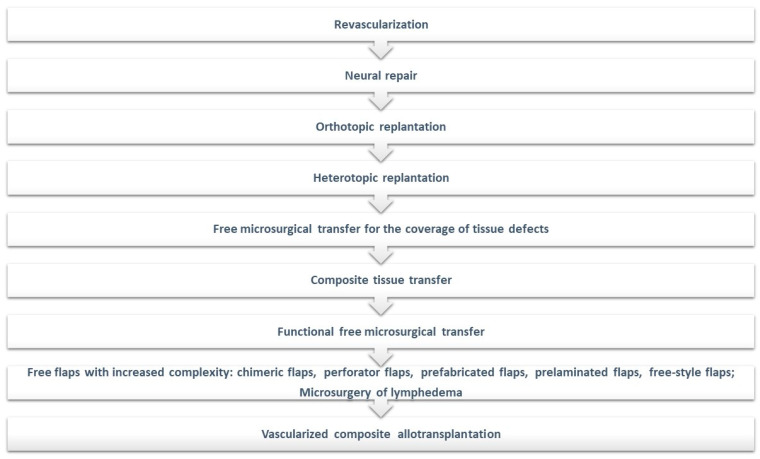
The updated microsurgical reconstructive ladder.

Since 1998, when the firsthand allotransplant was performed in France, the concept of vascularized composite allotransplantation gained popularity. This currently occupies the highest level on the microsurgical reconstructive ladder and will likely continue to do so with the advent of technological and immunological modern strategies. Figure 4 presents the microsurgical reconstructive ladder including all these new therapeutic strategies [12,102,108,113,114,115,116].

Once the idea of vascularized composite allografts was introduced as a new therapeutic entity, an emergent field within plastic surgery was created: the transplant reconstructive surgeon. Dr. Joseph Murray, trained as a plastic surgeon, was the first to perform a kidney transplant in 1954. He labeled the transplant procedure as the highest and most difficult intervention in reconstructive surgery. While conventional techniques achieve the reconstruction of tissue defects, the transplant “restores” structural and functional lost elements. A new paradigm occurred therefore, that of the “restoring” plastic surgeon, who must have complex technical abilities corroborated a good knowledge of biological mechanisms involved in transplantation. Approaching such reconstructive procedures involves patient integration within a broad, multidisciplinary program over a long time, in order to achieve the best results [117].

The field of vascularized tissue allotransplantation made possible the reconstruction of severe cases such as full facial loss, combined functional defects of the eyelids and perioral area, or severe loss of substance. To gain a better structural, functional and aesthetic outcome after VCA transplant, emerging techniques were associated mainly with head and neck reconstruction [118,119]. A study reported by Cho et al. evaluated a novel holographic craniofacial surgical planning application for use in facial transplantation, comparing its utility, cost, and limitations against magnetic resonance imaging and 3D printing technologies. Usually, computer-aided 3D modeling and 3D printed models have been used to improve the outcomes of such reconstructions by enabling virtual surgical planning. The research found that holographic models were more time and cost-efficient to produce than their 3D-printed counterparts. These holographic models offered detailed visualization of spatial relationships and allowed for virtual surgical planning and the virtual performance of face transplants through interactive manipulation of patient-specific anatomical holograms, efficiently and cost-effectively [120].

Knoedler et al. analyzed the possible integration of Artificial Intelligence (AI) into the domain of facial vascularized composite allotransplantation in order to address challenges in preoperative planning, the risk of malignancies due to immunosuppressive drugs, and the management of patient expectations due to the limited number of performed procedures. The paper identifies AI’s potential to enhance results in facial allotransplantation by offering precise outcome simulations, aiding in the diagnosis and prediction of rejection episodes, and enabling efficient malignancy screening [121].

Another challenging field is represented by the reconstruction of tracheal and laryngeal defects, which can be achieved, depending on the extent of the defect, through the use of prelamination and prefabrication methods alone or combined with allotransplantation techniques, as described by Vranckx et al [122]. Prelaminated radial forearm fascial flaps are utilized for short-segment tracheal defects. In cases of long-segment defects, a heterotopic prefabrication approach is adopted to ensure the vascularization of a tracheal allograft encased in forearm fascia, achieving chimerism by substituting the donor’s respiratory epithelium with the recipient’s buccal mucosa. Following the orthotopic transfer, this technique permits the gradual reduction and cessation of immunosuppression once bronchoscopy confirms the integrity of the trachea’s mucosa, as the recipient’s epithelium overtakes the foreign tracheal lining through a process of chronic rejection. The removal of a unilateral larynx tumor, often leading to total laryngectomy and loss of vocal cords due to the complexity of hemilarynx reconstruction, is addressed through a specific method: an autologous tracheal segment comprising four rings is first prefabricated using radial forearm fascia and then vascularized orthotopically to rebuild the laryngeal framework, aiming to save at least one vocal cord and thereby preserve the patient’s speech.

Orthotopic and heterotopic prelamination and prefabrication techniques present effective and reliable methods for repairing severe short and long-segment tracheal defects, as well as unilateral laryngeal defects [122]. Effective tracheal transplantation implies the use of tissue engineering techniques, employing constructs that do not generate an immune response, can revascularize rapidly, support the growth of epithelial tissue, and maintain mechanical characteristics similar to those of the original trachea [123].

As we can observe, the integration of vascularized composite allotransplantation into the field of reconstructive surgery, especially when combined with cutting-edge technological advancements, represents a transformative shift in our ability to address highly complex tissue defects. The association of VCA with emerging technologies, such as 3D printing, tissue engineering, and advanced imaging techniques, further enhances the precision and outcomes of these complex procedures. The synergy between VCA and these new technologies not only expands the limits of surgical possibilities, but also improves patient outcomes by reducing recovery times, enhancing functional rehabilitation, and ultimately improving the quality of life for individuals undergoing these complex reconstructive procedures.

## 3. Conclusions

The paradigms of plastic surgery went from traditional methods of reconstruction to complex models integrating a variety of decision-making factors. The concept has evolved in a way that it has to encompass all fields of reconstruction, taking into account etiology, the characteristics of the defect, the restoration of form and function, psychological aspects and the patient’s compliance, the surgeon’s skill and experience, as well as emerging technology. The increasing demand and appeal, the versatility of treatments offered and the refinements in the results have progressed tremendously. With the advent of innovative technologies, the field of plastic surgery has experienced significant development, making surgical procedures safer, more efficient and more patient-oriented than ever before. Therefore, one should bear in mind the aim of continuous improvement, refining established protocols in order to achieve the best reconstructive option for the patient.

## Data Availability

This paper is a literature review, no new data were created.
